# People's reports of unexpected events for everyday scenarios: Over 1000 textual responses, human-labelled for valence/sentiment, controllability and topic category

**DOI:** 10.1016/j.dib.2022.108545

**Published:** 2022-08-17

**Authors:** Molly S. Quinn, Courtney Ford, Mark T. Keane

**Affiliations:** a School of Computer Science, University College Dublin, Belfield, Dublin, Ireland; b Insight Centre for Data Analytics, University College Dublin, Belfield, Dublin, Ireland; c ML-Labs, SFI Centre for Data Analytics Research Training in Machine Learning, University College Dublin, Belfield, Dublin, Ireland

**Keywords:** Valence, Controllability, Explanation, Text data, Events, Sentiment

## Abstract

With this article, we present a repository containing datasets, analysis code, and some outputs related to a paper in press at *Cognition*. The data were collected as part of a pre-test, pilot test, and main study all designed in SurveyGizmo and participants recruited via Prolific.co (combined N=303). Datasets consist of raw and annotated data, where participant responses are free-text entries about what unexpected events might occur after a series of events, presented them with based on everyday scenarios. The code consists of all computational additions to the data, and analysis carried out for the results presented in the article. This data is released for the purpose of transparency and to allow for reproducability of the work. This human-labelled data should also be of use to machine learning researchers researching text analytics, natural language processing and sources of common-sense knowledge.

**Specifications Table**

**Table:** 

Subject	Psychology: Experimental and Cognitive Psychology
Specific subject area	This paper relates to a cognitive psychological study in which participants were asked to imagine unexpected events which might occur after the events describing everyday scenarios.
Type of data	Tables Figures Jupyter Notebooks
How the data were acquired	The data were collected via an experiment designed in SurveyGizmo and shared on the academic participant crowdsource platform Prolific.co.
Data format	Raw Analyzed Filtered
Description of data collection	Participants were recruited on the academic crowdsourcing platform, Prolific.co. Responses were collected via an experiment designed in SurveyGizmo. Responses were labelled by two independent raters, for which inter-rater reliability was measured, and (a small number of) disagreements were resolved by consensus.
Data source location	Country: United States of America, United Kingdom, Ireland
Data accessibility	Repository name: GitHub Data identification number: Direct URL to data: https://github.com/MollySQuinn/Control_and_Valence_in_Unexpected_Events
Related research article	M.S. Quinn & M.T. Keane, Factors affecting “expectations of the unexpected”; The impact of controllability & valence on unexpected outcomes. Cognition. (2022) 105142 https://doi.org/10.1016/j.cognition.2022.105142

## Value of the Data

• This data release supports the replication of the experiments (data collection and/or analyses) reported in Quinn & Keane (in press).
• The sharing of this data supports further insights into our understanding of unexpected events and event cognition in general.
• The data could be used for text analytics or machine learning applications dealing with expectations about event sequences or sentiments about events

## 1 Data Description

README.md – a markdown file that introduces the repository, the paper it represents, the data within, as well as how to run the code.

I. Folder: 0_data/

**Table:** 

Folder	File or Folder	Description
0_material_sets/	pre_test_material_sets/	Contains one file consisting of the material sets for each of the Latin-Graeco square condition sets defined in 1_code/1_Pre_Test.ipynb
	pilot_test_material_sets/	Contains one file consisting of the material sets for each of the counterbalanced conditions defined in 1_code/2_Pilot_Test.ipynb.
	materials_pilot_test_and_main_study.csv	The material set used in the pilot and main studies, with their corresponding mean perceived valence and perceived controllability scores from the pre-test.

1_pre_test_data/	0_raw_data/	The raw data collected from SurveyGizmo on 13 May 2020. Each file contains responses from 4 participants and corresponds to a Latin Graeco square and row (e.g.; LGs1r1 = Latin Graeco square one, row one) counterbalancing set (described fully in the next section). Contains participant ids, responses to each question (described in header), and some date/time information about the survey response.
	pre_test_data.csv	The filtered and annotated version of 1_pre_test_data/0_raw_data. Headers: material - material label, control - response to control question, q1 - response to attention check question (asks about the goal of material) valence - response to valence question, presentation_order – in which order the material subsets were presented (subsets described in PreTestMaterialSubsets.csv), condition_code - which condition was presented first (conditions described in ConditionAssignments.csv), subset - which subset the material belongs to, valence_condition - the intended valence condition (positive or negative), means_condition - the intended control condition (means present, means absent), goal_step, action_step, resources_step - these three columns are the presented sentences in the material scenario.

2_pilot_test_data/	0_raw_data/	The raw data collected from SurveyGizmo.
	1_annotated_data/	These are the raw data files from 2_pilot_test_data/0_raw_data with a column added to indicate the condition that was used in the survey that the raw data came from.

3_main_study_data/		Data files downloaded from SurveyGizmo on 15.09.2020. Two columns were added in manually: valence_condition - the intended valence condition (positive or negative) and means_condition - the intended control condition (means present, means absent).

II. 1_code

**Table:** 

File	Description
requirements.txt	The versions of python packages used to run the following files.
1_Pre_Test.ipynb	This file contains the analysis code and results used to choose the materials that met criteria to be used in furhter studies.
2_Pilot_Test.ipynb	This file contains exploratory analysis code for the few participants collected in the pilot test of the main experiment.
3_Expt1_Raw_Data_to_Labelling_Files.ipynb	This file contains the code used to change the raw data from 2_pipeline/materials_pilot_test_and_main_study.csv to files for labelling in 2_main_study/0_to_label
3_Labelled_Files_to_Kappa_to_Master.ipynb	This file checks the inter-rater reliability or agreement and prints the items that need to be agreed on by consensus. Once consensus is completed, the master data file is created here.
4_Experiment1_Analysis.ipynb	This file includes the output from all the 4x_Experiment1_... files, as well as some of its own analyses.
40_Intro_Expt1.ipynb	This file calculates the demographic information and sets up the functions for Chi-Square tests in the following notebooks.
41_Experiment1_Valence.ipynb	This file includes the analyses related to the outcome variable: valence of responses.
42_Experiment1_Control.ipynb	This file includes the analyses related to the outcome variable: controllability of responses.
43_Experiment1_ValenceXControl.ipynb	This file includes the analyses related to the interaction between outcome variables: valence of responses and controllability of responses.
williams_correction.py	This is a script that computes the William's correction for Chi-Square statistics and p-values given the frequency table “obs” and the Chi-Square statistic “chiobs”.

III. 2_pipeline

**Table:** 

Folder	File or Folder	Description
0_pre_test/	means_by_condition_ material.csv	This file contains the means and standard deviations for the perceived valence and perceived controllability for each version of each material.

1_pilot_test/	0_to_label/	These files have been created from the annotated files in 0_data/2_pilot_test_data/1_annotated_data/ for human labelling. There is one .csv file and one .xlsx file per material.
	1_MQ_labels/	These files have been manually labelled by rater MQ. Labelling was completed on 27th of July. There is one .csv file and one .xlsx file per material.

2_main_study/	0_to_label/	These files have been created from the annotated files in 0_data/3_main_study_data for human labelling. There is one .csv file per material.
	1_MQ_labels/	These files have been manually labelled by rater MQ. Copy of “2_pipeline/2_main_study/0_to_label” files for the rater. Responses randomized. A new column “random” was manually created and used to sort the spreadsheet. The order was the same for both raters. ID and unnecessary vars hidden. Category, valence, control, and goals label headings added. Labelling was completed on 27th of July. There is one .csv file per material.
	2_CF_labels/	These files have been manually labelled by rater CF. Copy of “2_pipeline/2_main_study/0_to_label” files for the rater. Responses randomized. A new column “random” was manually created and used to sort the spreadsheet. The order was the same for both raters. ID and unnecessary vars hidden. Category, valence, control, and goals label headings added. There is one .csv file per material.
	3_comparison_files/	This folder contains one csv file per material that shows the agreements and disagreements between the two raters MQ & CF.
	4_Final/	This folder contains the final agreements made after consensus on 2_pipeline/2_main_study/3_comparison_files.
	Labelling Criteria and Operational Definitions.docx	This document details the experimental design used to collect data, and operational definitions used to label collected data.
	master_data_codes.csv	This file contains the labelled data. Headers: user_id - participant user_id, response - text response of that user, ans_code - the Answer Category code/label, ans_count - a check that the user_id, response and label occur only once, val_code - the valence label, val_count - a check that the user_id, response and label occur only once, goal_code - the goal-word label (goal_object, non_goal_object, both_objects or
		neither_object), goal_count - a check that the user_id, response and label occur only once, control_code - the controllability label, control_count - a check that the user_id, response and label occur only once, material - material name that the response refers to.
	master_raw_data.csv	this data is downloaded from SurveyGizmo into 0_data/3_main_study_data and here is combined into one large dataset. Some demographic information (only that used in the Cognition article) are included.
	labels_and_descriptions.csv	This file maps the answer category code/label to a definition of that label.

IV. 3_output

○ control_report/ - The statistics and figures in the related paper were partly saved and loaded into Latex using a package called Kallysto. The following folders contain the data that was saved using this package.
  ▪ tex/
    • kallysto.tex - This is a latex file that lists the directories to each of the saved data and figures in 3_output/_kallysto/
  ▪ _kallysto/
    • data/

**Table:** 

Folder	File	Description
1_Pre_Test.ipynb/	PreTestMaterialSubsets.csv	These are the subsets materials were divided into in order to spread related themes (shopping, travelling, eating, etc.) evenly across counterbalanced sets.
	ConditionAssignments.csv	This table explains the Condition assignments.

3_Labelled_Files_to_Kappa_to_Master.ipynb/		Each text file contains the Cohen's Kappa inter-rater agreement for the category it is named for.

4_Experiment1_Analysis.ipynb/	Female.txt	percentage of females in the main study
	Ireland.txt	number of participants in the main study from Ireland
	Male.txt	percentage of males in the main study
	meanage.txt	mean age of participants in the main study
	N.txt	total number of participants in the main study
	stdage.txt	standard deviation around the mean age of participants in the main study
	UnitedKingdom.txt	number of participants in the main study from the UK
	UnitedStates.txt	number of participants in the main study from the USA

• defs/ - Each _definitions.tex file contains the latex definition of tables and data points to be included in the main tex file.
• figs/ - empty, but automatically generated folders
• logs/ - logs of kallysto being run

## 2 Experimental Design, Materials and Methods

The data and analysis code presented in this paper is related to a Cognition manuscript [1], and is split largely into three parts: the pre-test, pilot test, and main study. The following sections describe the experimental design, materials, and methodology.

### 2.1 Pre-test

The pre-test design relates to the 0_material_sets/pre_test_material_sets, 1_pre_test_data/* and 1_code/1_Pre_Test.ipynb files.

*Materials*: The pre-test was conducted to determine a set of materials for use in the main study. For the pre-test 4 versions of 20 materials (80 individual materials) were created. The 20 materials can be seen in the files in 0_data/0_material_sets/pre_test_material_sets/. Each of the twenty materials had 4 versions: Positively valenced and uncontrollable (means absent), positively valenced and controllable (means present), negatively valenced and uncontrollable (means absent), and negatively valenced and controllable (means present). Material versions were carefully matched on the objects introduced in the scenarios so that, to the best of our ability, the only things that differed between variants of the same material were the variables of interest, valence and controllability.

*Experimental Design*: The pre-test followed a Latin Square Design: 20 Materials x 2 Control (Present/Absent) x 2 Valence (Positive/Negative) x 2 Question Types (Control/Valence). The 20 materials were divided into four subsets of 5 materials each, deliberately chosen to divide materials with similar themes such as shopping or travelling equally into each subset.

The material subsets were then assigned to four different condition combinations by Control (Means Present, Absent) and by Valence (Positive/Negative).

The following are the Graeco-Latin squares used to counterbalance material subsets assignment to the four condition-combinations in the pre-test and main study. This design has been shown to remove both remote and immediate sequence effects where both condition order and material assignment should be counterbalanced [2].

Each row of each square corresponds to a condition set a participant could be assigned to. The letter refers to the condition combination seen in Table 2, and the number refers to the material subset seen in Table 1.

**Table 1:** Material subsets.

Subset 1	Subset 2	Subset 3	Subset 4
steve_gardening	rebecca_swimming	katie_kitten	sean_call
louise_shopping	sally_wine	lucy_loan	sam_driving
alan_plane	karen_bus	belinda_meeting	michael_tea
edith_exam	bob_job	peter_college	robert_essay
mary_food	bill_holiday	john_party	anna_interview

Material subsets found in PreTestMaterialSubsets.csv

**Table 2:** Condition combinations.

	Means Present	Means Absent
Negative	A	B
Positive	C	D

Condition combinations found in ConditionAssignments.csv

*Method*: Participants (N = 64) were randomly assigned to the counterbalanced subsets. Materials were randomly presented within their respective blocks. After each material, participants were asked to rate the controllability and valence of the scenario they just read on 7-point Likert-type scales. The Controllability and Valence questions were counterbalanced such that half of the participants in each Graeco-Latin square condition saw the controllability question first, and the other half saw the valence question first.

### 2.2 Pilot test methodology

The pilot test is related to the 0_data/2_pilot_test_data/*, 1_code/2_Pilot_Test.ipynb, and 2_pipeline/1_pilot_test/*. Latin Square Design: 8 Materials x 2 Control (Present/Absent) x 2 Valence (Positive/Negative).

*Materials*: The 8 materials (chosen from the pre-test) are divided into four subsets of 2 materials each deliberately chosen to divide materials with similar themes such as shopping or travelling equally into each subset (Tables  3 and 4).

**Table 3:** Graeco-Latin squares.

A1	C3	B2	D4	D2	B1	C4	A3
B3	A4	D1	C2	C1	D3	A2	B4
C2	D1	A4	B3	B4	A2	D3	C1
D4	B2	C3	A1	A3	C4	B1	D2

**Table 4:** Material subsets for pilot and main study.

	Subset 1	Subset 2	Subset 3	Subset 4
0	bill_holiday	rebecca_swimming	lucy_loan	sean_call
1	john_party	sally_wine	belinda_meeting	michael_tea

*Experimental design*: The material subsets were then assigned to four different condition combinations by Control (Means Present, Absent) and by Valence (Positive/Negative). Four material sets were created.

*Method*: Each material set was presented to a separate group of 5 participants. After each material, participants were asked to answer the question, “Something unexpected occurred. What do you think happened?” in a free-response text box. No participant saw more than one version of a given material. Materials were presented in a random order.

### 2.3 Main study

The main study is related to the files in 0_data/ 3_main_study_data, 1_code/4*, 2_pipeline/ 2_main_study, and 3_output.

The main study followed a similar design to the pilot test, using the same materials and design. A total of 219 participants were collected. Data from the main study were analyzed for the effects of material valence and material controllability on response valence and response controllability.

## Ethics Statements

Data collection from human subjects for all experiments listed was conducted with the approval of University College Dublin's ethics review board [LS-E-18-115-Keane-Exemption]. All participants completed informed consent before participating in the studies and were allowed to discontinue participation at any time.

## CRediT authorship contribution statement

**Molly S. Quinn:** Conceptualization, Methodology, Data curation, Formal analysis, Writing – original draft. **Courtney Ford:** Data curation. **Mark T. Keane:** Conceptualization, Methodology, Writing – review & editing, Supervision.

## Declaration of Competing Interest

The authors declare that they have no known competing financial interests or personal relationships that could have appeared to influence the work reported in this paper.

## Data Availability

People's Reports of Unexpected Events for Everyday Scenarios: Over 1000 Textual Responses, Human-Labelled for Valence/Sentiment, Controllability and Topic Category (Original data) (GitHub).
